# Going Alone

**Published:** 1864-01

**Authors:** 


### "GOING ALONE."

WITH curls in the sunny air tossing,
With light in the merry blue eyes,
With laughter so clearly outringing,
A laugh of delight and surprise;
All friendly assistance disdaining,
And trusting no strength but its own—
The past fears and trials forgotten,
The baby is "going alone."

What woeful mishaps have preceded
This day of rejoicing and pride!
How often the help that he needed
Has carelessly gone from his side!
He has fallen while reaching for sunbeams,
Which, just as he grasped them, have flown,
And the tears of vexation have followed,
But *now* he is "going alone."

And all through his life he will study
This lesson again and again;
He will carelessly lean upon shadows,
He will fall, and weep over the pain.

The hand whose fond clasp was the surest
Will coldly withdraw from his own,
The sunniest eyes will be clouded,
And he will be walking alone.

He will learn what a stern world we live in,
And he may grow cold like the rest,
Just keeping a warm sunny welcome
For those who seem truest and best;
Yet, chastened and taught by past sorrow,
And stronger and manlier grown,
Not trusting *his all* in their keeping,
He learns to walk bravely alone.

And yet *not* alone, for our Father
The faltering footsteps will guide
Through all the dark mazes of earth-life,
And "over the river's" deep tide.
Oh! here is a Helper unfailing,
A strength we can perfectly trust,
When, all human aid unavailing,
"The dust shall return unto dust."

